# Novel antifouling paint formulation based on Ca_2_​Cr_2_O_5_​ and CaMnO_3_​ NPs as a protective pigment

**DOI:** 10.1038/s41598-024-74245-3

**Published:** 2024-10-18

**Authors:** H. Abd El-Wahab, Hossa F. Al-Shareef

**Affiliations:** 1 Chemistry Department, Faculty of Science, Al-Azhar University, Nasr City, Cairo 11884 Egypt; 2 Department of Chemistry, Faculty of Science, Umm Al-Qura University, Makkah, Saudi Arabia

**Keywords:** Antifouling paint, Protective coating, Mixed metal oxide NPs pigments, Corrosion resistance, Chemistry, Materials science

## Abstract

This work focused on the preparation of novel antifouling paint based on Ca_2_Cr_2_O_5_ and CaMnO_3_ NPs as a safe protective pigment which were replaced with cuprous oxide. Three paint formulations were prepared for comparison, a blank formula without an antifouling agent (F1), a commercial antifouling formula based on 100% cuprous oxide as an antifouling agent (F2), and AF formula based on 75% Ca_2_Cr_2_O_5_ and CaMnO_3_ NPs and 25% Cu_2_O. The high performance and durability of the paints based on the prepared pigments were evident from their impact resistance, adhesion, pending, hardness, and chemical resistance, which were compared to the blank formula (F1). The corrosion resistance of the painted films was also investigated using the salt spray test method, and the results were promising compared to the blank and standard formulations. All painted steel plates were exposed to seawater through field tests in the Suez Canal at Port Said for up to 6 months. The results showed that the paints based on F2 and F3a, b enhanced the antifouling activity through six months of exposure. The obtained results demonstrated greater efficiency of the painted steel-based F3a than F1 and F3b, and being comparable to the standard formula (F2).

## Introduction

The undesirable growth of microbes, algae, and crustaceans on submerged, exposed substrates is known as marine biofouling. Both dynamic and static structures are severely impacted by fouling (vessel speed reduction, increased fuel consumption, increased hull maintenance, etc.). Many techniques have been employed in the lengthy history of fouling avoidance, including copper sheathing, pitch, and tar. Therefore, to protect coated surfaces from marine microorganisms, paint formulas that have historically included biocidal species are employed^1,2^.

The goal of antifouling is to inhibit or stop the growth of organisms on underwater surfaces. Antifouling agents prevent pollutants from adhering to surfaces, eliminate microorganisms that come into contact with the surface, and stop the growth of microorganisms and the creation of biofilms. A subclass of antifouling materials with biocidal properties is known as an antimicrobial or antibacterial agent. Much research has been done on materials with antifouling and antibacterial qualities in order to develop antifouling surfaces for biomedical implants and devices^3,4^.

The earliest antifouling paints date back to the mid-19th century and were made with toxicants like some metals such as Cu_2_O, dissolved in shellac or drying oil. However, tributyltin (TBT)-based antifouling paints have proven to be the most effective antifouling paints in terms of long-term efficacy. Since January 1, 2008, these TBT-based products have been completely banned due to environmental concerns. In the past ten years, modified coatings-based polymer matrices occasionally combined with rosin—and different types of biocides that come into contact with fouling organisms have emerged as contemporary antifouling solutions^5^. These consist of mineral substances like cuprous oxide and, less frequently, cuprous thiocyanate, as well as chemical molecules known as booster biocides. In general, booster biocides are toxic to aquatic life^6^. The two most common active ingredients in modern antifouling coatings are natural biocides and cuprous oxide. Because cuprous oxide is prone to bioaccumulation, it should always be replaced by more environmentally friendly mixtures. Conversely, cuprous oxide is not only an antifouling agent; it is also an essential component in the antifouling coating’s leaching and surface finishing processes. Sr, Ca, Zn, and Mg peroxides are assessed for use as pigments in antifouling coatings. Highly saltwater-soluble metal ions and H_2_O_2_ are produced when the oxidizing agents react with seawater^7,8^. In the marine industry, biofouling is a persistent issue that calls for substantial financial resources for control and novel cleaning techniques. The production of environmentally acceptable, low-toxicity, and harmless antifouling compounds is urgently needed for maritime firms and underwater equipment, as marine coatings based on trichlorophenol (TBT) were outlawed worldwide in 2008^9^. Creating a formula based on natural substances or new biocides (like medetomidine and econea) would be another antifouling tactic^6,10,11^. Bellotti et al. have demonstrated the potential of zinc “tannate” antifouling paints; nevertheless, antifouling efficacy depended on the formulation, as the antifouling activity was altered by the matrix and plasticizer used^12^. Antibacterial properties are exhibited by several inorganic nanoparticles based on some metal oxides. Their mechanism includes the release of metal ions (Ag and Cu) and the generation of active oxidative stress (TiO_2_ and ZnO) in response to UV radiation^13,14^. It is particularly desirable to have membrane surfaces with multiple defensive mechanisms because of the variety and complexity of membrane fouling. It may be possible to stop biofilm growth and fouling with one of the most promising materials for modifying membrane surfaces. NPs and polymers provide microbial and fouling resistance properties, respectively, and polymer nanocomposites have been produced^15,16^. Self-polishing copolymer (SPC) coatings remain the most widely used commercial antifouling products. However, they are now mainly made of copper acrylate. These coatings function similarly to the efficient copper-based TBT coatings, offering an acrylate copolymer that hydrolyzes quickly and refreshes the surface, as well as a leaching element for hydrolysis. The potential for continually employing copper has expanded recently with the emergence of copper pyrithione and the development of copper NPs^17^. Because copper relied on a comparable mode of action for biocide release and offered a similar level of effectiveness in a variety of settings, it was able to replace TBT. Most of the potential mechanisms for copper’s antibacterial effect have to do with either disrupting or penetrating a cell membrane^18–21^. By manipulating its size and shape, copper’s antifouling activity can be adjusted. The distinctions between copper’s nano, micro, and macro forms were examined by Chapman et al.^21^. They discovered that the coatings on copper nanoparticles (NPs) absorbed the least amount of protein, carbs, and slime when suspended in either a sol-gel or polydimethylsiloxane (PDMS), followed by microparticles and, finally, bulk copper^22^. Fe, Cr, and Co-metal complexes based on hydroxy acetophenone benzoyl hydrazone were reported to have antimicrobial and antifouling properties. By submerging the coated films in seawater, the produced metal complexes combined with epoxy resin were tested for durability, antibacterial activity, and antifouling properties. The results showed that Fe, Cr, and Co-metal complexes might be used as antifouling materials^23^.

Various heterocyclic compounds were prepared and tested for their biological activity against macrobiofoulants and their suitability as environmentally friendly biocides in a particular kind of self-polishing paint that contained no tin. The findings demonstrated that the prepared compounds’ biocidal, antimicrobial, and antifouling activities were highly significant, suggesting that they could be employed as antifouling agents^24^. The design techniques and advancements of chemically hybridized polymer–ceramic hybrid antifouling coatings, including a step-by-step hybrid strategy and a one-step sol-gel hybrid strategy, were presented. Moreover, the mechanical and antifouling properties of stereoscopic polysiloxane structures are explored. It looked at what percentage of organic and inorganic components, as well as how crosslinking was done, could indicate the next generation of protective antifouling coatings^25^. The formulation of rosin paint against marine microorganisms using mixed metal oxide nanoparticles is examined for the first time. After that, they are tested and added to an antifouling paint. We will look at a preliminary assessment of the interest in focusing on microfouling to achieve effective paint.

## Experimental

### Materials

All chemicals and reagents used in the experiment were of analytical grade. It is unnecessary to purify these chemicals further before use since they could be used as received from the supplier, which were obtained from multiple chemical companies (Merck, Alpha Chem, Fluka, Loba, and El Nasr Company, respectively).

### Preparation of calcium–chrome, and calcium–manganese oxides, (Ca_2_Cr_2_O_5_, CaMnO_3_,) NPs

Calcium–chrome, and calcium–manganese oxides, (Ca_2_Cr_2_O_5_ and CaMnO_3_), were prepared by using the co-precipitation method. A solution of calcium carbonate (CaCO_3_), chromium carbonate (Cr_2_(CO_3_)_3_) in a calculated 1:1 molar ratio, was prepared. Drops of dilute nitric acid (HNO_3_) were added to reach complete dissolution and clear solution. A slight amount of residual contaminants was removed by filtration. Then the filtrates were added slowly to a magnetically stirred solution of ammonium carbonate ((NH_4_)_2_CO_3_). Upon addition of the salt solutions, various precipitates with different colors are formed. After complete additions of the salt solutions, the stirring was continued additional time to ensure of complete precipitation and homogeneity of the products. Then the precipitates were filtered out of the residual solution and dried. The dried precipitate of mixed-phase carbonates was then fired at 900 ^0^C to give Ca_2_Cr_2_O_5_. The same procedure was applied for synthesizing CaMnO_3_^26,27^.

### Instrumentation

The phase compositions and bond structures of various prepared samples were determined from the X-ray diffractograms (XRDs) utilizing X-ray diffraction analysis (XRD) data using a (Bruker D8 advance instrument, Germany). Copper Kα radiation with a wavelength of 1.54 Å was used over a 2θ range of 20°–80° at room temperature. Fourier transform infrared (FTIR) spectra were recorded on (Bruker, Vector 22 single-beam spectrometer, Germany) with a resolution of 4 cm^−1^. The samples were ground with KBr (in a 1:100 ratio) to form tablets, which were then mounted in the spectrometer’s sample holder. Measurements were recorded at room temperature in the range of 400–4000 cm^−1^. Chemical compositions of selected powder samples were analyzed using an ICP instrument (Leeman Labs Inc., Profile Plus 2004, USA). A scanning electron microscope (SEM, JEOL JSM-T 330 A) with an acceleration voltage of 30 kV was used to study the morphology of the precipitated powders. The prepared pigments were evaluated for their oil absorption (ASTM D281-12(2021), hydrogen ion concentration (pH value) (ASTM D1293-12), bleeding of pigments, ASTM D279-02(2019), degree of fineness: ASTM D1210-05(2022), moisture content: ASTM D2216-19, and loss on ignition (ASTM D7348-21).

### Microbial biofouling assay

#### A. Test solutions

The same concentration of each of the Ca_2_Cr_2_O_5_ and CaMnO_3_ NPs were prepared. Tested concentrations were 100–10,000 mg/L for each one at different time intervals: 0 times, 24 h, 48 h, 72 h, and 96 h.

#### B. Mussels

Samples of adult Brachidontes variabilis marine mussels were taken from the waters of the Suez Gulf at Attaqah Mountain. The collected mussels were maintained in 70 cm × 40 cm × 40 cm glass aquariums filled with seawater. Constant aeration, twice-weekly water changes, and occasional removal of dead bivalves were provided. In each experiment, adult mussels ranging in size from 0.5 cm to 1.0 cm were employed.

Ten mussels were used in each test, and the test materials were added to 1-litre beakers containing 500 millilitres of saltwater to reach final concentrations of 100–10,000 mg/L. The same procedures were followed when applying control samples. However, no chemicals were examined^28,29^.

### Paint compositions

The paints were prepared using a high-steering mixer at the beginning of the procedure, followed by using a laboratory ball mill to incorporate the prepared pigment NPs and other solid materials. Preparation of the paint formulations were based on gum rosin and vinyl resin as binders, with prepared mixed metal oxide NPs and/or cuprous oxide as antifouling pigments. The formulations also included additives such as a dispersing agent, plasticizer, and epoxy (low molecular weight as additive) to create a tin-free antifouling paint formulation, using the same procedure as for the preparation of the blank sample (F), according to the pigment volume concentration, **P/B:2:1**. The paints were then applied to steel and glass panels using a brush. All efforts were made to maintain a uniform film thickness of 100 ± 5 μm. The composition of the paint formulations is tabulated in Tables 1, 2, 3^24^.

**Table 1:** Paint formulation free biocide, (F1).

Composition	Percentage
Alkyd resin	27.69
TiO_2_	23.00
Kaolin	21.62
Iron oxide	12.40
Dispersing agent	0.25
Plasticizer	1.70
Xylene	13.34

**Table 2:** Antifouling paint formulation based on cuprous oxide as a biocide, (F2).

Composition	Percentage
Xylene	18.54
Gume Rosine	15.40
Cuprous oxide	40.00
Epoxy low Mwt = 700	00.19
Dispersing agent	00.25
Plasticizer	01.70
Iron oxide	6.40
Zinc phosphate	04.80
Talc	7.62
Vinyl resin	05.10

**Table 3:** Antifouling paint formulation based on prepared mixed metal oxide (F3).

Composition	Percentage
Xylene	18.54
Gume Rosine	15.40
Cuprous oxide	10.00
Prepared mixed metal oxide	30.00
Epoxy low Mwt = 700	00.19
Dispersing agent	00.25
Plasticizer	01.70
Iron oxide	6.40
Zinc phosphate	04.80
Talc	7.62
Vinyl resin	05.10

#### Procedure of painted steel plates

Four panels, each measuring 25 cm by 40 cm by 1 mm, were prepared for testing; one will be utilized as a blank and the other as a test panel. After oil and grease were removed using solvent cleaning as per SSPC-SP1, the panels underwent abrasive blasting according to ISO 8501-1 to achieve a Sa 2.5 finish. The surface roughness, measured with the Micrometer Elcometer 124 and the Replica Tape Elcometer 122, was 75 μm. The surface was roughened using sandpaper. The panels were then cleaned with fresh water and painted with 100 ± 5 μm of the prepared AF paint^29^.

### Physical and mechanical properties of the coated specimens

The paint films underwent a range of mechanical and physical analyses. Steel panels were prepared according to ASTM D609-17. Film thickness was measured using ASTM D1005-13. Film hardness was determined using a pencil hardness tester following ASTM D3363-11, and specular gloss measurements were carried out according to ASTM D523-18. Flexibility was assessed using ASTM D522-17, and adhesion was tested with a cross-hatch cutter following ASTM D3359-17. The resistance of organic coatings to rapid deformation (impact) was evaluated using ASTM D2794-93 (Reissued in 2001). Corrosion resistance was tested according to ASTM B117-19.

## Results and discussion

### Infrared spectra

**Fig. 1:**
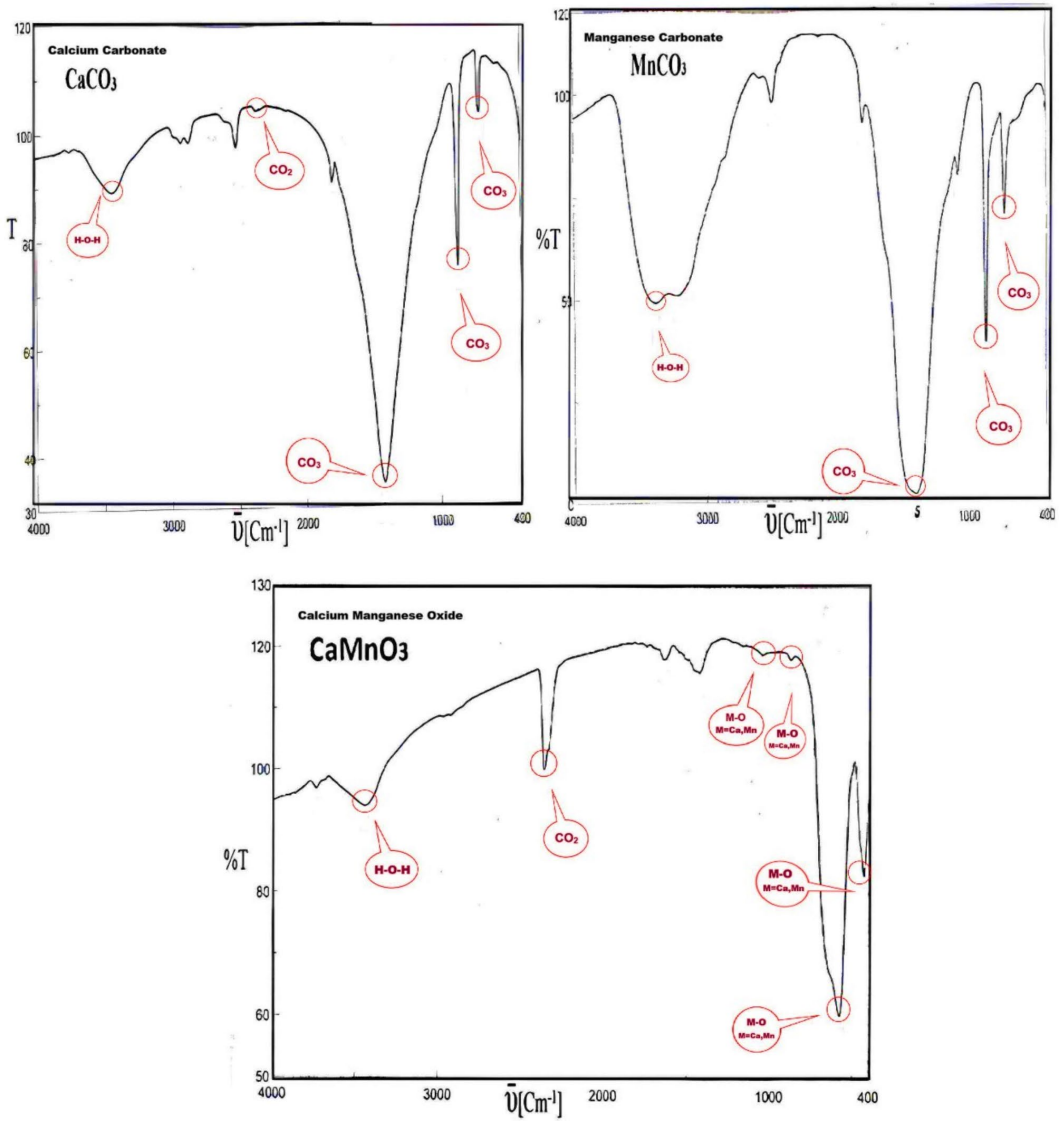
FT-IR of CaMnO_3_, and corresponding CaCO_3_, MnCO_3_^26^.

**Fig. 2:**
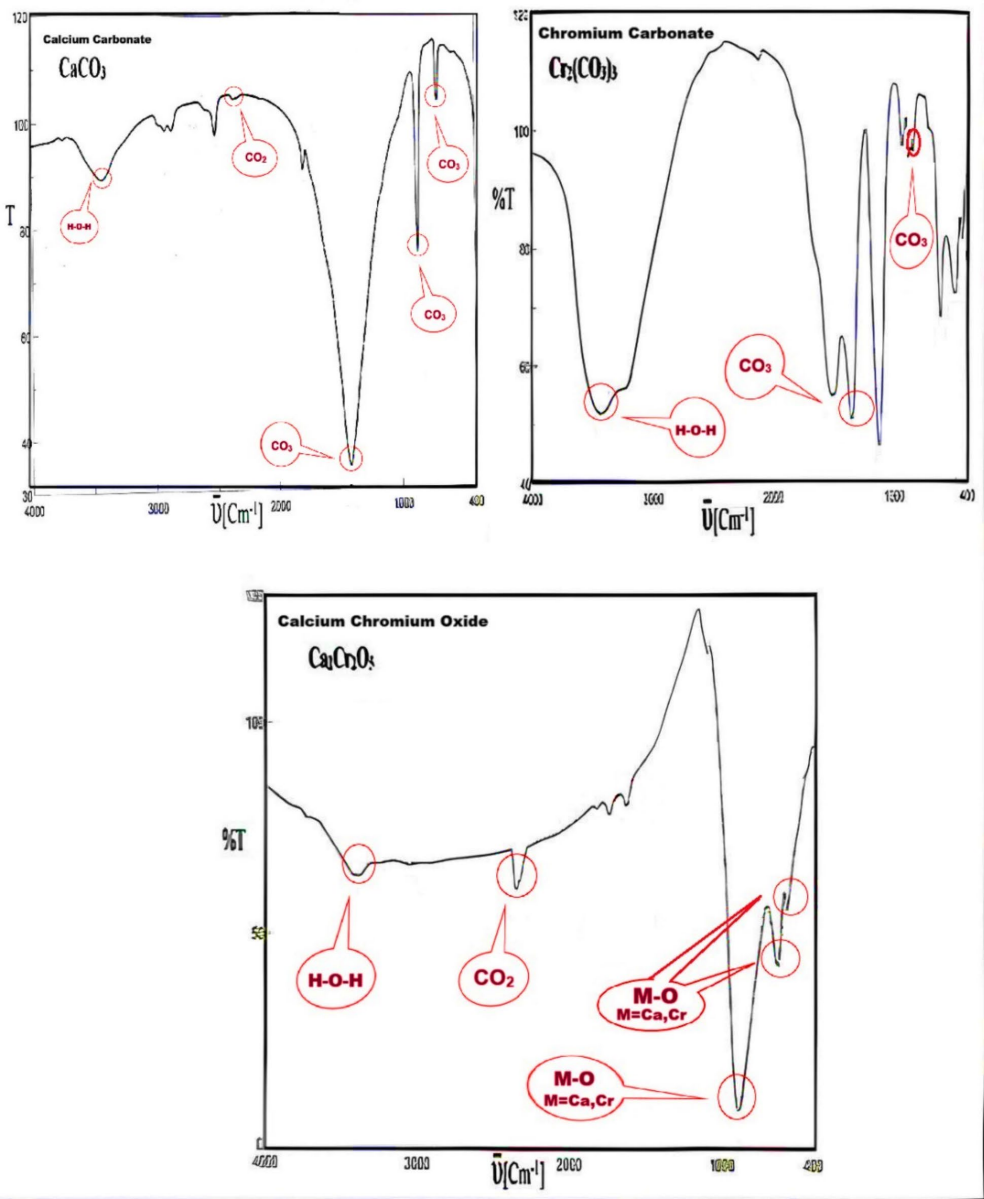
FT-IR of Ca_2_Cr_2_O_5_, and corresponding CaCO_3_, Cr2(CO)_3,_^26^.

The prepared mixed metal oxide NPs were confirmed by FTIR and XRD, according to our previous work, and the figures of FTIR are presented in Figs. 1 and 2, and Tables 4 and 5^26,27^.

**Table 4:** IR spectra of CaMnO_3_, CaCO_3_ and MnCO_3_.

CaMnO_3_			CaCO_3_			MnCO_3_		
υ (cm^−1^)	Intensity	Assignment	υ cm^−1^	Intensity	Assignment	υ cm^−1^	Intensity	Assignment
3439	W	H-O-H	3435	Broad	H-O-H	3384	Broad	H-O-H
2360	M	CO_2_	2364	VW	CO_2_	1395	Broad	υ3 CO_3_
1045; 1427; 871; 576; 429	W, VW, S, S, Broad	M-O (M = Ca, Mn)	1425	VS	υ3 CO_3_	862	VS	υ2s CO_3_
			874	S	υ2s CO_3_	718	S	υ4as CO_3_
			707	M	υ_4as_ CO_3_			

**Table 5:** IR spectra of Ca_2_Cr_2_O_5_ and CaCO_3_ and Cr_2_(CO_3_)_3_ .

Ca_2_Cr_2_O_5_			CaCO_3_			Cr_2_(CO_3_)_3_		
υ (cm^−1^)	Intensity	Assignment	υ (cm^−1^)	Intensity	Assignment	υ (cm^−1^)	Intensity	Assignment
3395	Broad	H-O…H	3435	Broad	H-O-H	3425	Broad	H-O…H
2361	W	CO_2_	2364	VW	CO_2_	1348	S	υ2s CO_3_
1634	W	δ O-H	1425	VS	υ _3_ CO_3_	839	M	υ _4as_ CO_3_
891; 639; 578	S, M, M	M-O (M = Ca, Cr)	874	S	υ _2s_ CO_3_			
			707	M	υ _4as_ CO_3_			

Infrared spectra provided information on the nature of the bonding structures in the prepared mixed oxide samples. Tables 4 and 5, summarize the data obtained from FTIR spectra analysis of the synthesized sample products. The obtained infrared spectrum from CaCO_3_ (Tables 4 and 5) revealed the presence of very strong absorption band at 1425 cm^−1^ which corresponding to **υ**3 and strong absorption band at 874 cm^−1^ attributed to **υ**2 symmetric vibration of CO_3_ and a characteristic absorption band at 707 cm^−1^ for **υ**4 asymmetric vibrations of CO_3_. In addition, the broad absorption bands averaged at 3435 cm^−1^ and the very weak band at 1642 cm^−1^ are assigned to the stretching and bending vibration of H_2_O, respectively. It is worth mentioning that the two absorption bands which are characteristic of H_2_O are detected in all infrared spectra of the products. The disappearance of the vibrational absorptions is characteristic of the carbonate ions at 1450, 1395 and 1348 cm^−1^ ; and 890–820 cm^−1^ corresponding to **υ**2 and **υ**4 (symmetric and asymmetric) for CaCO_3_, MnCO_3_ and Cr_2_(CO_3_)_3_, respectively. New spectra absorption bands that appear to be associated with newly formed species consistent with this is the appearance of new absorption bands at 1427, 1045, 576, and 429 cm^−1^ due to different modes of M-O (M = Ca, Mn). The absorption bands at 8,91,639 and 578 cm^−1^, as shown in Table 5, of Ca_2_Cr_2_O_5_, can also be ascribed to M-O bonds (M = Ca, Cr)^27^.

### X-ray diffraction analysis

**Phase identification and crystal structure**

Understanding the crystal structure and phase purity of CaMnO_3_ and Ca_2_Cr_2_O_5_ are crucial for optimizing its performance in various applications. X-ray diffraction (XRD) is one of the primary techniques used to analyze the crystallographic structure of materials like CaMnO_3_., and Ca_2_Cr_2_O_5_. Once synthesized, the sample is ground into a fine powder to ensure uniformity and then placed in an XRD instrument. The XRD pattern is obtained by directing X-rays onto the sample and measuring the intensity of diffracted rays as a function of angle (2θ). The intensity and position of these peaks can be compared with standard reference patterns from databases such as the Joint Committee on Powder Diffraction Standards (JCPDS). A well-defined set of sharp peaks indicates high crystallinity and phase purity, while broad or additional peaks may suggest impurities or secondary phases.

**Fig. 3:**
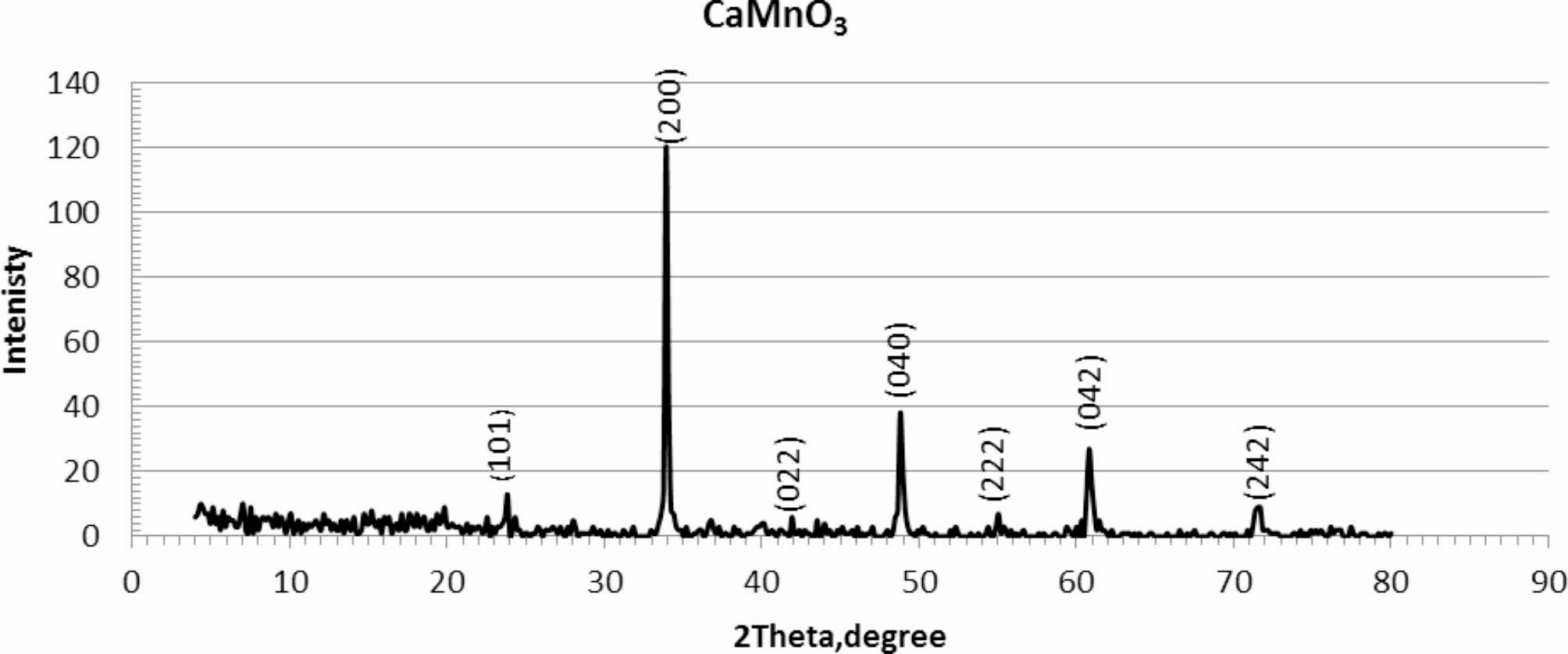
XRD patterns of the prepared CaMnO_3_ mixed oxide^26^.

Figure 3 shows the X-ray diffraction pattern of the prepared MMO sample from calcination at 900 °C for 2 h of the precursors obtained from co-precipitation of CaCO_3_ and MnCO_3_ powders. Copper Kα radiation with a wavelength of 1.54 Å was used over a 2θ range of 20–80° at room temperature. It reveals the formation of a perovskite structure. All XRD peaks are assigned to the calcium manganese oxide (CaMnO_3_) phase and have a high crystalline structure and high compatibility with (JCPDS #50-1746). So, based on the data available from JCPDS card the comprehensive analysis reveals that the significant 2-theta values for XRD analysis of CaMnO_3_ include those at approximately: 2θ = 25°, 35°, 43°, 50°, 55°, 362°, and 73°. which correspond to Miller Indices in (101), (200), (022), (040), (222), (042) and (242), planes, respectively. The crystal lattice structure constant was found in good agreement with the standard reported.

**Fig. 4:**
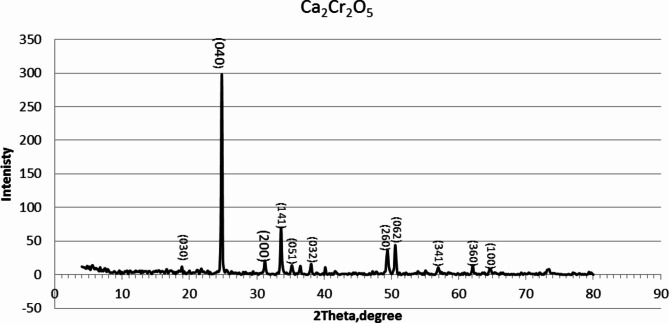
XRD diffraction patterns of Ca_2_Cr_2_O_5_ mixed oxide^26^.

Figure 4 shows the XRD pattern of the prepared Cr_2_(CO_3_)_3_. All XRD peaks are assigned to the dicalcium dichromium oxide phase and have a high crystalline structure and high compatibility with JCPDS (48–0791) card. From JCPDS card 48–0791, we can extract several key 2-theta values associated with Ca_2_Cr_2_O_5_. These values represent angles at which constructive interference occurs due to X-ray scattering from different planes within the crystalline structure. The data available from JCPDS card 48–0791, here are some notable 2-theta values for Ca_2_Cr_2_O_5_: 2-theta values for XRD analysis of Ca_2_Cr_2_O_5_ include those at approximately: 2θ = 20°, 25°, 33°, 35°, 37°, 39°, 49°, 52°, 58°, 63°, and 65°. which are correspond to Miller Indices in (030), (040), (200), (141), (051), (032), (260), (062), (341), (360), and (100) planes, respectively. The crystal lattice structure constant was found in good agreement with the standard reported^30–35^.

### Scanning electron microscope (SEM) of the prepared CaMnO_3_ and Ca_2_Cr_2_O_5_

**Fig. 5:**
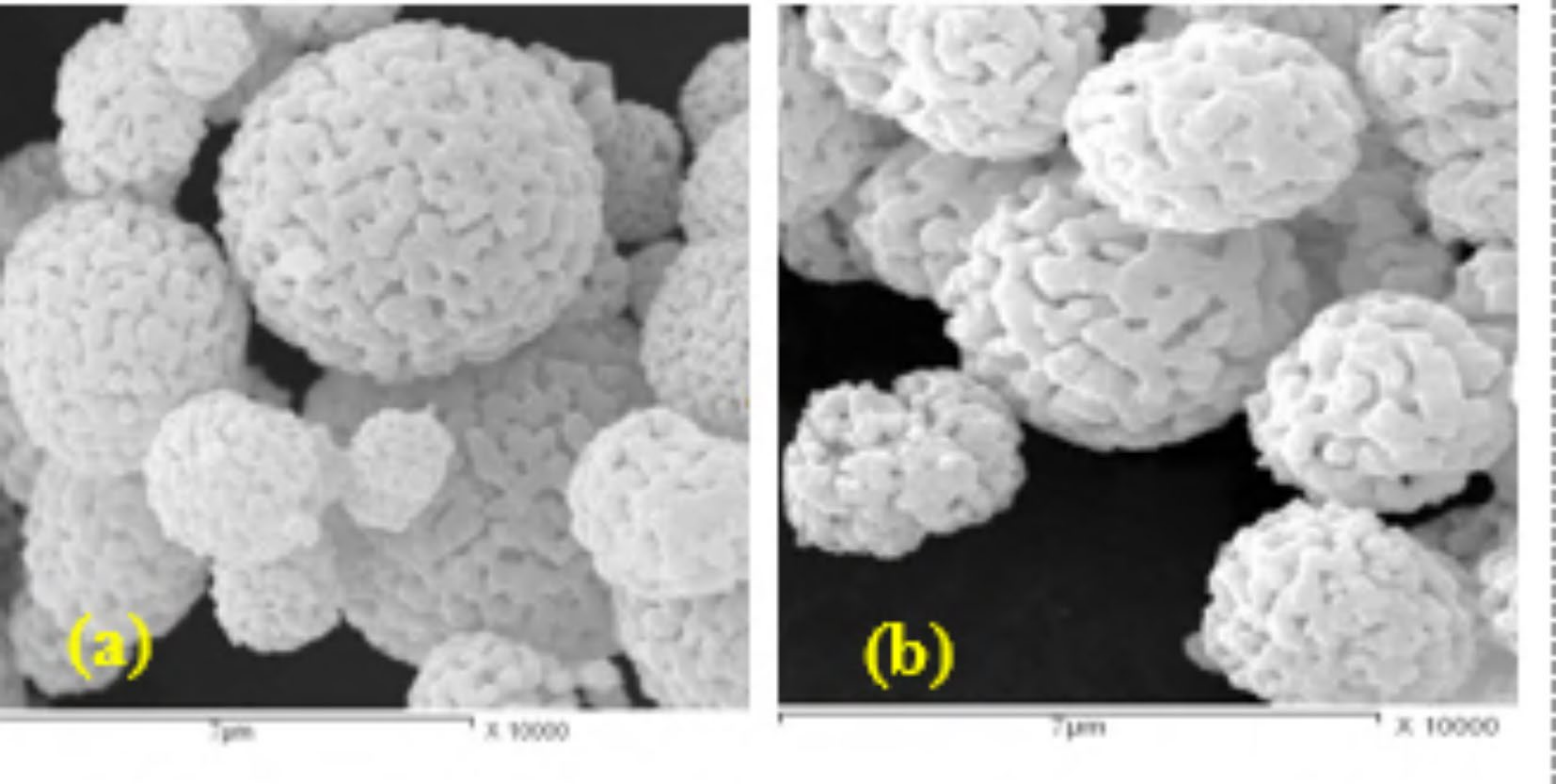
The SEM images of (**a**) Ca_2_Cr_2_O_5_ NPs and (**b**) CaMnO_3_ NPs.

Figure 5a,b show the SEM images of the precipitated powder samples. Figure 4a,b shows the SEM image of the CaMnO_3_ sample which appears as spherical in nature and the spherical particles of good hiding power to other surfaces. Whereas the porous structure of the spheres is good for absorption of oil. The size of the particles is in the range of nano size. The spherical structure of the prepared samples increased the oil absorption percentage which is an important factor for oil absorption for computability with bined in the paint formulation.

### Transmission electron microscopy (TEM) of the prepared CaMnO_3_ and Ca_2_Cr_2_O_5_ NPs

**Fig. 6:**
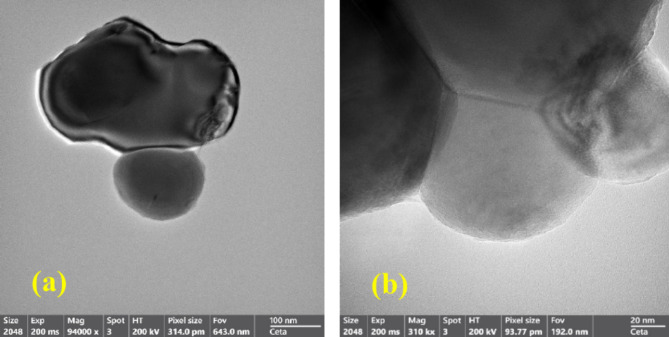
The SEM of (**a**) Ca_2_Cr_2_O_5_ NPs and (**b**) CaMnO_3_ NPs.

Figure 6 shows Transmission Electron Microscopy TEM) images of the CaMnO_3_​ and Ca_2_​Cr_2_O_5_​, (a, b), nanoparticles show a somewhat spherical morphology with an particle size of about 20 nm of CaMnO_3_​ (a), and 100 nm of Ca_2_​Cr_2_O_5_​, this is an important factor for oil absorption, enhancing compatibility with vehicles.

### ICP analysis

ICP (Inductively Coupled Plasma) Spectroscopy is an analytical technique used to measure and identify elements within a sample matrix based on the ionization of the elements withing the sample.

Table 6 presents the results of the ICP measurements of the samples and suggests that the discrepancy between the measured and calculated metal concentrations in the calcined samples and prepared mixtures may be due to the differing deposition preferences of the calcium ions. Ca ions tend to deposit in an alkaline medium, whereas manganese (Mn) and chromium (Cr) ions are more readily deposited in an acidic medium.

**Table 6:** Inductive coupling plasma results.

Products	Theoretical	Practical
CaMnO_3_	Ca = 27.9% Mn = 38.46%	Ca = 21.67% Mn = 37.48%
Ca_2_Cr_2_O_5_	Ca = 30% Cr = 39%	Ca = 16.27% Cr = 24.36%

### Evaluation of the prepared pigment (mixed metal oxide NPs)

The ASTM measurements of the pigment properties listed in Table 7 revealed the following: Where: T = toluene; E.G., = ethylene glycol; B.A. = butyl glycol; N.B. = normal butanol; M.E.K. = methyl ethyl ketone; L.O.I. = loss on ignition; H = Hagman (fineness unite). We can observe the following results based on the tabulated results in Table 7.

**Table 7:** Physico-chemical characteristics of the prepared mixed metal oxide NPs.

Products	L.O.I	pH	Oil absorption	Moisture	Fineness	Bleeding							
						T	EG	BA	*N*.B	MEK	CH_2_Cl_2_	CHCl_3_	CCl_4_
Ca_2_Cr_2_O_5_	0	10.8	50	0.6	8H	None	None	None	None	None	None	None	None
CaMnO_3_	0	11.0	114	0.8	8H	None	None	None	None	None	None	None	None

The ASTM measurements of the pigment properties listed in Table 7 revealed the following: T = toluene, E.G. = ethylene glycol, B.A. = butyl glycol, N.B. = normal butanol, M.E.K. = methyl ethyl ketone, L.O.I. = loss on ignition, and H = Hagman (fineness unit). Based on the tabulated results, we can observe the following in Table 7.

### pH values: ASTM D1293-12

The obtained values for the prepared (Ca_2_Cr_2_O_5_ and CaMnO_3_) NPs ​were slightly alkaline, which is beneficial for resisting the salinity of seawater during microbiofouling test.

### Oil absorption: ASTM D281-12(2021)

Oil absorption is a well-known indicator of whether a pigment will consume binder when applied to paints. More binders will be required to fully wet the pigment and create a homogeneous paint film as the oil absorbs more paint. Table 7 illustrates that calcium-chromium oil absorption was the lowest in the group, while calcium-manganese oil absorption was the highest.

### Moisture content: ASTM D2216-19

The moisture results of the two prepared mixed metal oxide nanoparticles are very low, indicating a negligible effect of moisture on the weight of the pigment before use in the paint formulation.

### Degree of fineness: ASTM D1210-05(2022)

Based on the results in Table 7, the degree of fineness for the prepared CaMnO_3_ and Ca_2_​Cr_2_​O_5_​ was observed to be 8H for Ca_2_Cr_2_​O_5_​ NPs and 7H for CaMnO_3_​. This indicates that the higher efficiency of results based on Ca_2_Cr_2_O_5_​ is due to its better dispersion in the pigment-vehicle compared to CaMnO_3_​.

### Bleeding of pigments: ASTM D279-02(2019)

This test method measures the percentage of color produced when the pigment comes into direct contact with various solvents. It is useful as a quick and simple test for the pigment’s overall bleeding properties. The observed results show a high degree of stability for the prepared mixed metal oxide NPs, with Table 7 indicating no bleeding (non-dissoluble color).

### Loss on ignition: ASTM D7348-21

This test calculates the pigment weight loss during high-temperature ignition. The results show that the pigment does not change in weight or color when subjected to high temperatures, indicating that the created mixed oxides are incredibly stable under various conditions.

### SEM of the paint formula based on CaMnO_3_ and Ca_2_Cr_2_O_5_

**Fig. 7:**
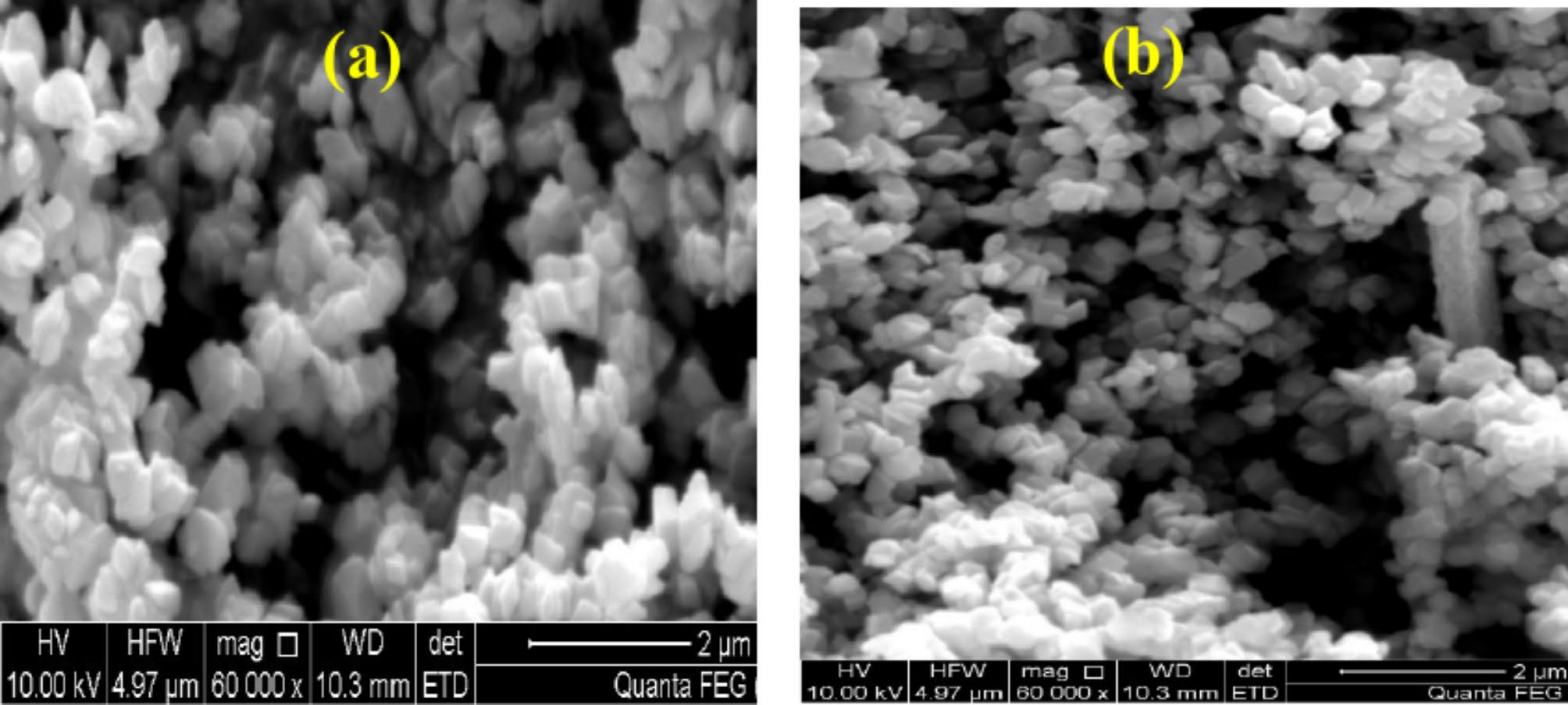
SEM images of the paint formula based on CaMnO_3_ (**a**) and (**b**) Ca_2_Cr_2_O_5_ NPs.

The SEM images of the paint formulation based on F3, which contains CaMnO_3_ ​and Ca_2_​Cr_2_O_5_​, illustrate that there are no morphological abnormalities in this formulation, as shown in Fig. 7. This is consistent with the pigment-vehicle dispersion of both CaMnO_3_​ and Ca_2_​Cr_2_O_5_​, suggesting good dispersion. However, it is possible that Ca_2_Cr_2_​O_5_​ was dispersed similarly to CaMnO_3_ NPs. This aligns with the fineness of grind and oil absorption results reported for both manufactured metal oxide NPs.

### Mechanical properties and chemical resistance of painted films

**Fig. 8:**
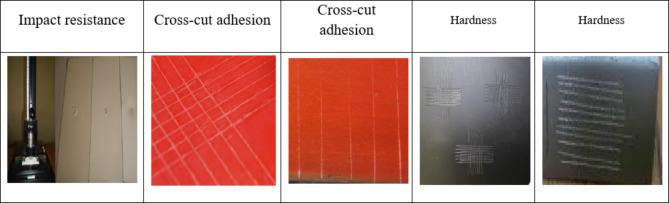
Shows the tested mechanical characteristics such as impact, cross-cut adhesion, and hardness of the coated steel films.

As listed in Table 8 and shown in Fig. 8, which represent the mechanical properties and chemical resistance of dry-painted films based on all paint formulations (F1, F2, and F3a, b). From this table, it can be concluded that all painted films have passed the chemical resistance test with no observable changes detected in the samples. At the same time, the recorded results of the mechanical properties of the tested films showed an improvement in impact resistance (**1.3–1.9 kg**), scratch hardness (**1.4 to > 2.2 kg**), and adhesion cross-cut (**4B-5B**) for the dry-painted films of F1 to F3a, b, respectively. This improvement in mechanical properties and chemical resistance for the painted films based on Ca_2_​Cr_2_O_5_​ and CaMnO_3_ NPs was observed and showed that paint-based mixed metal oxide NPs are more efficient than samples based on F1 and are comparable to paint-based F2 (A.F. commercial sample). This efficiency may be attributed to the presence of protective pigments in nanosized form, which helps achieve high dispersion in the pigment-vehicle mixture, enhancing performance and durability. This can also be attributed to the elastic properties of the two types of polymers used (Gum rosin with vinyl resin), and the integration of Ca_2_​Cr_2_O_5_​ and CaMnO_3_ NPs into the cavities of gum rosin and vinyl resin can hinder any defects that may lead to damage.

**Table 8:** Mechanical properties and chemical resistance of dry painted films.

Composition	Impact (kg)	Adhesion	Pending	Scratch hardness (kg)	Dry film thickness	Acid	Alkali	Solvent and water
Paint formula, F1, (sample blank)	1.4	4B	Pass	1.4	100 ± 5 μm	Pass	Pass	Pass
Paint formula (F2) based on Cu_2_O	D = 1.6	5B	Pass	1.7	100 ± 5 μm	Pass	pass	Pass
Formula based on Ca_2_Cr_2_O_5_ with [F3a]	1.7	5B	Pass	> 2.2	100 ± 5 μm	Pass	Pass	Pass
Formula based on CaMnO_3_ [F3b]	D = 1.9	5B	Pass	> 2	100 ± 5 μm	Pass	Pass	Pass

### Evaluation of corrosion resistance

**Fig. 9:**
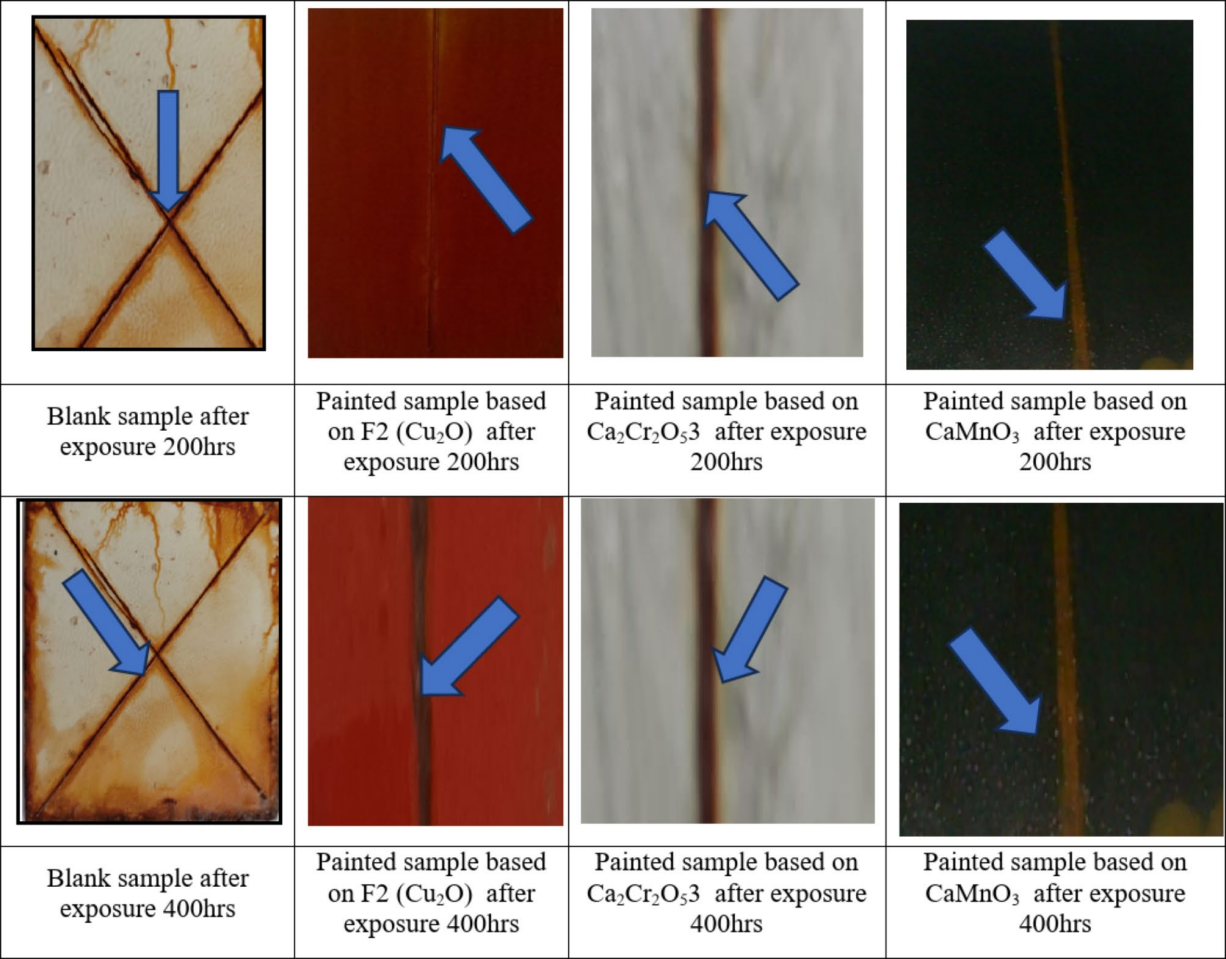
Photographic of corrosion resistance of the painted steel panels.

The anticorrosion performance of the paint formulations on the metal surface due to exposure to a saltwater environment was examined using a salt spray test. After testing, the analyzed samples were compared with control panels, and images from the salt spray test are shown in Fig. 9. When these images were analyzed, it was observed that the sheet plates coated with the blank sample (F1) did not show effective anticorrosion performance, failing to protect against corrosion. While there was minimal blistering, rusting increased over time, starting after 200 h and becoming more pronounced by 400 h. In contrast, the coatings based on cuprous oxide as in F2, and those based on the prepared Ca_2_Cr_2_​O_5_​ NPs and CaMnO_3_ as in F3a and F3b, exhibited higher resistance to corrosion. According to ASTM B117-19, the corroded surfaces were analyzed, and the results related to corrosion failure are presented in Table 9. The failure was evident on the panel coated with F1, but was almost unnoticeable on the coatings that contained the prepared mixed metal oxides as in F3a and F3b, as well as those based on cuprous oxide (F2), after 400 h of exposure. It was observed that the corrosion resistance of the tested paint panels increased compared to the panel coated with F1. However, surface deterioration of the panels coated with cuprous oxide and CaMnO_3_​ started after 400 h. This improvement in corrosion resistance can be attributed to several factors:

(i) The use of the prepared mixed metal oxide increased the surface hardness and hydrophobicity.
(ii) The use of the prepared mixed metal oxide decreased the coating porosity. This reduction effectively prevented the diffusion of Cl^**-**^ ions into the polymer-coated metal plates, thereby extending the coating’s ability to protect against corrosion for an extended duration.
(iii) The presence of the prepared mixed metal oxide NPs and their good dispersion within the gum rosin and vinyl resins resulted in improved adhesion to the substrate, acting as a barrier to isolate mild steel from corrosion. This barrier is impermeable to water and corrosive ions, reducing water permeability.
(iv) The long chains in the gum rosin structure made the resin more hydrophobic, reducing water holding power and improving corrosion protection.
(v) The interface between the two resins (gum rosin and vinyl resin) increased the crosslinked network, which acts as an insulating layer that hinders the transport of electrons from the metal surface to aggressive materials necessary for rust formation. Additionally, they act as barriers that slow down the propagation of aggressive species and corrosion products^36^.
(vi) Sometimes, the failure of the coating layer starts when spots or holes form due to aging or mechanical shocks. Aggressive and corrosive materials can then attack the underlying surface through these holes, leading to an increase in exposed area and accelerating the corrosion process. These materials can easily react with the substrate, eventually causing anodic or cathodic delamination. The presence of both resins can form a tough layer that prevents this delamination,^26,27,36,37^.

**Table 9:** Evaluation of corrosion resistance of the painted films.

Formulation No.	Blistering		Scribe failure (mm)	Rust grade
	Size	Frequency		
Paint formula free biocide (F1)	5	MD	4	5
Paint formula (F2) based on Cu_2_O	7	F	2	7
Formula based on.Ca_2_Cr_2_O_5_ with [F3a]	8	F	2	9
Formula based on CaMnO_3_ [F3b]	7	F	2	7

### Evaluation of the efficiency of paints against microfouling

**Fig. 10:**
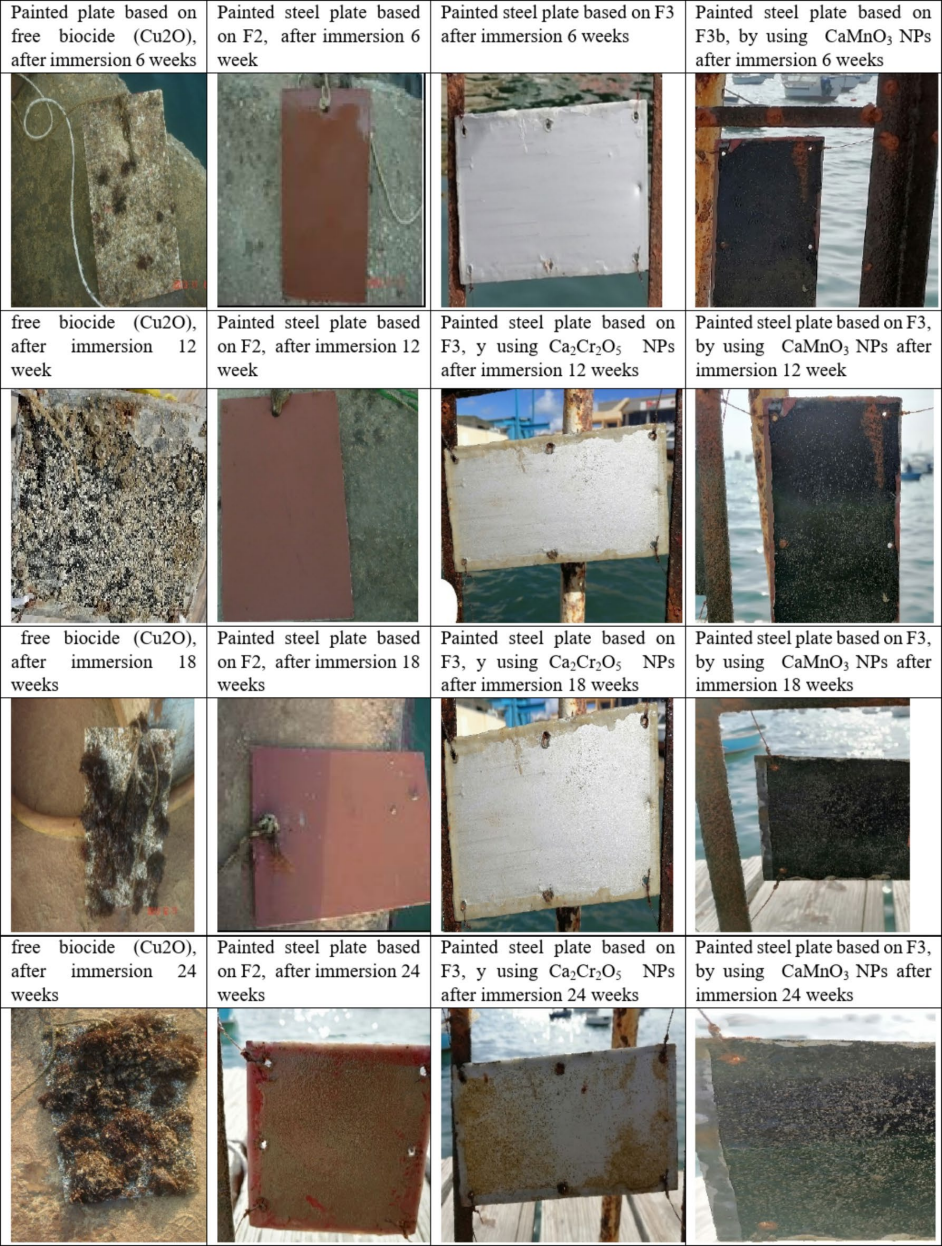
Shows the photos of the painted steel plates after immersion in seawater, (**a**) painted with free antifouling, (**b**) painted plate without incorporated with metal oxide NPs, (**c**) painted plate based on Ca_2_Cr_2_O_5_ NPs (**d**) painted plate based on CaMnO_3_.

The ability of antifouling paints to prevent fouling organisms from attaching or growing, along with their durability, adhesion, smoothness, and ease of application, is essential for their effective function. Table 3 illustrates an experiment using a vinyl resin matrix. Since mixed metal oxide NPs are commonly included as pigments in many paints, it is crucial to examine how they impact the formulation’s performance. This research primarily focuses on the effect of substituting Cu_2_O in the formulation with mixed metal oxides. It has been demonstrated that substituting 75% of Cu_2_​O with the prepared mixed metal oxide NPs resulted in new antifouling paint formulations, as shown in Table 3, based on F3a and F3b. Compared to the commercial formulation containing 40% cuprous oxide as a biocide and antifouling agent (AF), the results of these trials regarding proper pigmentation indicate that the new formulations provide better pigmentation outcomes. Therefore, replacing 75% of the cuprous oxide in dry-painted steel plates with the prepared mixed metal oxide NPs enhances performance and durability, including corrosion resistance. The paint formulation F3 was designed to account for the matrix solution rate, which determines how quickly toxic particles are exposed to seawater. The marine coating systems were exposed to seawater for approximately six months (twenty-four weeks) from January to June 2023 in the Suez Canal (Ismailia, Egypt). The efficiency of the paints was evaluated by visual inspection of macrofouling on the coatings after six months of immersion. The results of anti-microfouling activity during the first weeks of immersion are shown in Fig. 10. Micrographics revealed that steel plates painted with F2 and F3 were efficient against microfouling, similar to those coated with the antifouling formula containing biocide (F2). Based on the investigation and the visual photographs shown in Fig. 8, the following conclusions can be drawn:

**After 8 weeks** of immersion in seawater, the painted steel plates based on F1 (free biocide formula) exhibited significant fouling, with the development of homogeneous and thicker fouling films due to the growth of microalgae on the surface. A thick and dense biofilm was observed^38^. In contrast, no wet weights or settlement were recorded on the painted steel plates based on F2 and F3, which were more efficient against micro biofouling. This effectiveness is attributed to the Cu_2_​O in F2, which is more resistant to fouling and microorganisms, and the presence of prepared CaMnO_3_ and Ca_2_​Cr_2_​O_5_ NPs in F3, which also contributed to preventing settlement and growth of macrofouling and enhancing the high durability of the paint.

**After 16 weeks** of immersion, the fouling covered approximately 100% of the area on the painted steel plates based on F1 (free biocide formula), which were completely fouled. In contrast, the plates based on F2 and F3, particularly those with Ca_2_​Cr_2_​O_5_ NPs, remained more efficient and highly resistant to various types of fouling. The painted steel plates based on F3 (CaMnO_3_​) showed some settlement and growth of macrofouling but still maintained antifouling activity.

**After 18 weeks** of immersion, significant antifouling activity was still noted on the painted steel plates based on F2 and F3. However, the plates based on F3 (CaMnO_3_) NPs showed some settlement and growth of macrofouling. The wet weights of fouling on these substrates were observed, compared with painted plates based on F2 and Ca_2_Cr_2_​O_5_ NPs, which still exhibited significant antifouling activity and were not significantly affected due to the presence of Cu_2_​O in F2 and metal oxide NPs in F3.

**After 24 weeks** of immersion, the fouling covered approximately 100% of the painted steel plate F1 area, which was completely fouled. The main biofouling organisms observed included various algae resistant to the coating layer, leading to failure of the coating system and extensive settlement from other fouling organisms. As seen on the painted steel based on F3 (CaMnO_3_​) NPs only showed slime and a few green algae during the six months of immersion. The painted plates based on F2 and Ca_2_​Cr_2​_O_5_ NPs still demonstrated significant antifouling activity, with no accumulations or settlements recorded, due to the coatings’ hard, smooth surfaces, which limited the adhesion strength of foulants. A significant issue with using Cr and Mn metal oxide NPs as antifoulants is their potential to increase corrosion resistance when applied to steel surfaces, similar to other mixed metal oxide NPs investigated previously as anticorrosive pigments^24,37^.

### Proposed mechanism of antifouling paint against biofouling

The type of biocide was considered when designing three paint formulations: F1, which is a biocide-free paint; F2, a commercial antifouling standard based on cuprous oxide (F3a); and F3b, which is based on CaMnO_3_​ NPs and uses gum rosin and vinyl resin as binders. These paints are listed in Tables 2 and 3. Tin-free AF paints (F2 and F3) generally have a longer service life due to their ability to slow down the release rate of the binder, which helps prevent biofouling from growing and settling^39^. Additionally, as part of the antifouling paint mechanism to prevent biofouling adhesion, seawater penetrates the paint matrix, dissolves biocides and co-biocides, and allows other additives to slowly diffuse back into the bulk paint^39^. Depleted primary biocides, such as Cu_2_​O or the prepared mixed metal oxide NPs, form a thin layer of leached antifouling paint^39^. There are two types of paint leaching releases in self-polishing polymers (gum rosin and vinyl resin): early leaching and steady-state leaching^39–41^. Table 2 (F2) shows the elemental composition of copper as the primary biocide in AF paint F2, while Table 3 (F3a and F3b) lists Cr and Mn in F3. The high concentration of Cu indicates the presence of primary biocide compounds (Cu_2_O) in the AF paints (F2). The high resin levels are influenced by the presence of a high concentration of chloride ions in seawater, which increases the dissolution rate of Cu_2_​O^41^. When seawater interacts with cuprous oxide, soluble hydrated Cu(I) chloride complexes are formed. During the hydrolysis process, Cu^2+^ can replace Cu^+^ as the primary biocidal species^42^. Chemical reactions and diffusion processes, including the dissolution of seawater-soluble pigments, binder reactions, and paint polishing, regulate the biocide release rate^43^. Conversely, the high concentration of chloride ions in seawater does not affect the Ca_2_​Cr_2_​O_5_ and CaMnO_3_ NPs used, which may impact the consistency of the leached layer in self-polishing antifouling (SPC-AF) paints^24,37^. This suggests that the polymer matrix retains tiny holes due to the absence of biocides, increasing the paint’s overall wetted area. The leached layer undergoes hydrolysis, changing the binder’s wettability from hydrophobic to hydrophilic^44,45^. The self-polishing effect of partially reacted binders can occur when exposed to less-reacted paint surfaces due to erosion by streaming seawater. A matrix enhanced with Cu or Cr, Mn, and biocide on less-reactive paint surfaces further prevents marine biofouling from adhering. The antifouling results also, indicated that the incorporation of the prepared mixed metal oxide NPs into coatings enhanced the antifouling performance of the coating by improving the coating hydrophobicity and decreasing the coating elastic modulus^46–48^.

## Conclusion

Due to their tiny size, nanomaterials have different characteristics from conventional materials in terms of protection. Recently, mixed metal oxide NPs have gained recognition as powerful reactive anticorrosive pigments. Based on the findings, it can be concluded that the optimal pigment loading in gum rosin and vinyl resin-based paints is 2:1% by pigment volume concentration (PVC). So, the mixed metal oxide NPs were evaluated as biocides in a tin-free, self-polishing antifouling paint., by preparation of Three paint formulations (F1 as a primer without any biocide), F2 (antifouling formulation based on 100% cuprous oxide) and F3 based on 75% Ca_2_​Cr_2_O_5_​ and CaMnO_3_ instead of cuprous oxide (F3 a, b). All the painted films were tested according to the corrosion resistance and against slime-forming micro biofouling organisms. The results showed that the tested formulations’ corrosion resistance properties and their antifouling agents’ behavior followed, F2 ˃ F3a > F3b > F1. This promising performance result of the prepared paint formulations based on mixed metal oxide NPs can be attributed to several critical factors. First, A lower toxicity for these active substances was revealed comparatively to cuprous oxide, Second, many recent corrosion inhibitors are based on metal oxides, particularly mixed metal oxides, and finally, due to their effectiveness against antimicrobial activity.

## Data Availability

Data is provided within the manuscript or supplementary information files.
